# Twenty Years in California

**Published:** 1869-11

**Authors:** 


### TWENTY YEARS IN CALIFORNIA.

Twenty years ago to-day the steamer "Oregon," R. H. Pierson, captain, Richard Whiting, first officer, landed about four hundred Pioneers in San Francisco, of which five were females. Of that number a few yet remain in the State, five or six of whom are prominent citizens of San Francisco. The writer, who was personally acquainted with all, remembers about twenty as still living in different parts of California, half of whom, perhaps, have realized, to a satisfactory extent, their object in visiting the land of gold. A few others made moderate fortunes and went East. Many more have died, including the two chief officers of the "Oregon." It is a remarkable fact that not one of these four hundred Pioneers has been sent to the Penitentiary or to Congress, and only a few even to the Legislature.—*San Francisco Bulletin, September* 18.

---

"Cultivate not only the corn-fields of the mind, but the pleasure-grounds also," was a motto of Dr. Whately's.

There is a voice from the tomb sweeter than song; there is a remembrance of the dead to which we turn, even from the charm of the living.

Honest industry is always rewarded. No young man need complain of being kept poor, if he rolls up his sleeves, and goes cheerfully to work.

